# Accurate conventional and microwave-assisted synthesis of galloyl hydrazide

**DOI:** 10.1016/j.mex.2019.11.010

**Published:** 2019-11-15

**Authors:** Amgad M. Rabie

**Affiliations:** Department of Pharmaceutical Organic Chemistry, Faculty of Pharmacy, Mansoura University, Mansoura 35516, Egypt

**Keywords:** Abs., absolute, ACN, acetonitrile, Aq, aqueous, Arom., aromatic, Conc., concentrated, Conv., conventional, DEE, diethyl ether, EtOAc, ethyl acetate, EtOH, ethanol, MeOH, methanol, M.P., melting point, MS, mass spectrometry, MW, microwave, MWI, microwave irradiation, M.Wt., molecular weight, Pet., petroleum, Recryst., recrystallized, Rel. Int., relative intensity, Rotavap, rotary evaporator, RP, reversed-phase, R.T., room temperature, S, singlet, Str., strong, T.D.S., triple distribution system, TFA, trifluoroacetic acid, TMS, tetramethylsilane, V/v, volume per volume, V/v/v, volume per volume per volume, Xss., excess, **Chemical Transformation** (esterification and hydrazinolysis reactions, synthesis using both conventional heating and one-pot solventless greener microwave heating techniques) followed by **Complete Purification and Full Elucidative Characterization** (chromatographic, physicochemical, spectroscopic, and elemental characterization for identification), (Compounds: **3,4,5-Trihydroxybenzohydrazide (galloyl hydrazide**, target compound, coded as **2nz**) and **Ethyl 3,4,5-trihydroxybenzoate (ethyl gallate**, intermediate compound, coded as **1nz**), 3,4,5-Trihydroxybenzohydrazide, Galloyl hydrazide, Ethyl gallate, Microwave-assisted synthesis, Spectroscopic analyses

## Abstract

The usefulness of the structure of galloyl hydrazide (3,4,5-trihydroxybenzohydrazide, coded as **2nz** in this article) as a privileged structural system in pharmaceutical organic and medicinal chemistry has prompted the advances of the further therapeutic potentials of this known antioxidant/antitumor compound and, in addition to that, it acts generally as a very important organic reaction intermediate for molecule planning (including synthesis of many important biologically active molecules), as it undergoes various types of chemical reactions. The data of the two synthetic methods (including the new green one), presented in this research article, provides sharp adequate chemical data about the challenging synthesis, separation (purification), and characterization of this compound. A new and very fast one-pot solventless greener microwave-assisted method of synthesis, in addition to the much slower old conventional one, is used in this present research; and followed by full precise purification and characterization (including chromatographic separation; physicochemical identification; IR, ^1^H-NMR, ^13^C-NMR, and mass spectroscopic analyses along with elemental analyses for structure elucidation) of **2nz**.

• Galloyl hydrazide is a very important and unique organic chemical intermediate with, mainly, antioxidant and anticancer biological activities.
• Galloyl hydrazide synthesis in the previous literature is very challenging, confusing, not standardized, and without any consensus.
• The main objective of this new research is to make two standard and fixed methods of synthesis (conventional and greener) of galloyl hydrazide through designing and constructing a new one-pot solventless greener microwave-assisted synthetic method, in addition to redesigning and qualifying the old traditional conventional heating one; both methods are followed by very accurate identification and characterization data (which can be referenced for the efficient reproducibility in the organic and pharmaceutical chemistry community) of galloyl hydrazide.

## Graphical abstract

**Figure:**
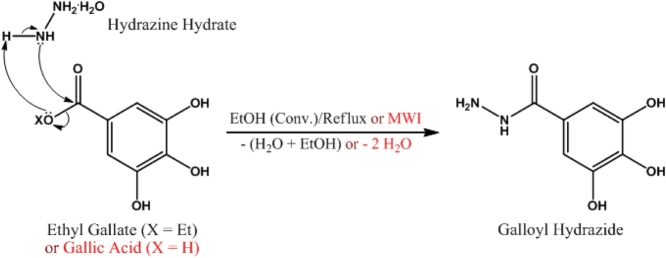

**Specification Table**

**Table:** 

Subject area:	Organic Chemistry followed by Analytical Chemistry, Physical Chemistry, and Spectroscopy (Data Category: Synthesized, Chromatographic, Physicochemical, and Spectral)
More specific subject area:	Conventional and Microwave-assisted synthesis; Thin-layer chromatography (using Ultraviolet light detection) and High-performance liquid chromatography; Recrystallization, Yield, Color, Appearance, and Melting point; IR, ^1^H-NMR, ^13^C-NMR, and Mass spectroscopic analysis; Elemental analyses (Data Type: Analyzed (in the article text) and Raw (in the article figures of the scanned photocopies and the accompanying supplementary files of the research data))
Method name:	Chemical Transformation (esterification and hydrazinolysis reactions; synthesis using both conventional heating and one-pot solventless greener microwave heating techniques) followed by complete purification and full elucidative characterization (chromatographic, physicochemical, spectroscopic, and elemental characterization for identification) (Compounds: **3,4,5-Trihydroxybenzohydrazide** (**galloyl hydrazide**, target compound, coded as **2nz**) and **Ethyl 3,4,5-trihydroxybenzoate** (**ethyl gallate**, intermediate compound, coded as **1nz**))
Name and reference of original method:	Various tens of unfixed and confusing procedures in the literature (for the Conventional Method) and Completely new procedure (for the Microwave Method)
Resource availability:	Detailed resources (Instruments and Procedures) specifications and resulted Data are included within this article (all used equipments are ordinary and basic chemistry laboratory equipments, and very easily available)

## Method details

### Rationale

**Scheme 1:**
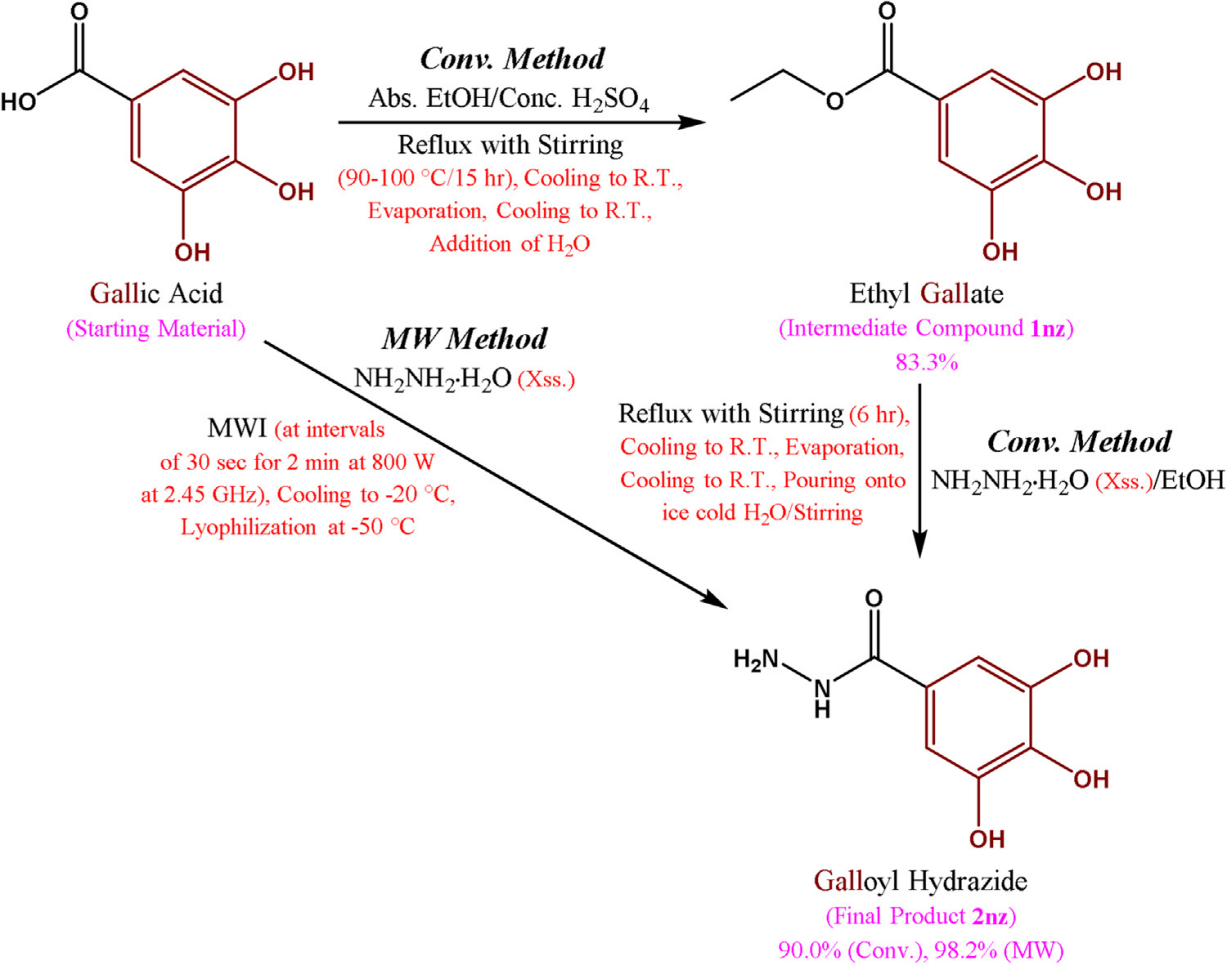
Synthetic route for the synthesis of the hydrazide **2nz**.

Galloyl hydrazide (3,4,5-trihydroxybenzohydrazide, **2nz**) is a very important chemical intermediate in organic synthesis, which is, mainly, well-known for its antioxidant and antitumor bioactivities [[1], [2], [3], [4], [5], [6], [7], [8], [9]]. The main objective of this new research is to make two (conv. and greener) standard methods of synthesis of **2nz** through designing and finding a new one-pot solventless greener MW-assisted synthetic method, in addition to redesigning and qualifying the old traditional conv. one; followed by very accurate identification and characterization data which can be referenced for the efficient reproducibility in the organic chemistry community. As illustrated in Scheme 1 (below), the new benign MW-assisted procedure starts with gallic acid (3,4,5-trihydroxybenzoic acid) which undergoes hydrazinolysis in just one very fast step to directly give **2nz** in 2 min without passing through the very slow esterification reaction; unlike the much slower two-step conv. procedure which comprises first the synthesis of the intermediate ethyl gallate (ethyl 3,4,5-trihydroxybenzoate, **1nz**) through esterification of gallic acid, which is followed by another slower step of the hydrazinolysis reaction of **1nz** to form **2nz**. By using the present new chemical data collection, the organic/medicinal chemists and drug designers/discoverers would have the following apparent advantages in the synthesis of **2nz**:

1. Avoiding the clear differences among most of the previous literature procedures for the synthesis of **2nz** through providing two fixed accurate methods (conv. and benign) of synthesis (supported with complete extrapurification technique) followed by constant and reliable reproducible data of physicochemical properties and spectroscopic/microanalytical analyses of **2nz**.
2. Using the easily synthesized ester of gallic acid, the ethyl ester **1nz**, which is the most common ester (ethyl esters are the most common and known esters of almost all carboxylic acids), unlike most other literature methods which use the less common propyl ester of gallic acid [7,[10], [11], [12], [13], [14], [15], [16]] (the ethyl ester **1nz** was preferred in the present work over the famous propyl gallate ester, i.e., it is the ester of choice for this hydrazinolysis reaction, due to many reasons, such as providing the reaction with an internal autosolvent through giving EtOH as a byproduct of the reaction, which could be condensed, recovered, and reused as a solvent for the same reaction mixture, so less volume of the primary original EtOH would be used leading to reduced reaction costs, unlike the propyl ester which would give propanol instead; also ethyl group is a much better leaving group than the longer propyl group in this reaction, because it is easier to leave than the propyl one which is heavier than it, so a faster reaction with better yield would be obtained; and, in addition to these advantages of using it over the propyl gallate, the ethyl ester is mostly the first choice for this type of reactions).
3. Designing a newer and greener method of synthesis of **2nz** (MW-assisted procedure), which is much more preferred than the conv. heating method (see Table 1, shown later), because it has/is:
  a. Decreased number of synthetic steps (just one direct step).
  b. Extremely decreased heating/reaction time (very rapid synthesis of just 120 sec).
  c. Much more benign and environmentally safe (green MW procedure).
  d. Extremely more economic and much cheaper (due to many reasons, e.g., only one inexpensive starting material, carboxylic acid, with only one reagent are needed in just one step).
  e. Highly efficient (due to many reasons, e.g., well-enhanced reaction rate, the hydrazide is produced with a yield of 98.2% and a purity of about 99.0%).
  f. Solventless (no solvents used at all).
  g. Self-catalyzed as there is no catalyst needed (used) at all.
  h. Much less energy consumption (extremely minute amount of 0.02667 KWhr is only needed).
  i. Higher overall yield of the hydrazide (98.2%) and with excellent purity.
  j. Decreased waste production (just one step of one reactant and one reagent giving only one final product with a very high yield of 98.2% and an excellent purity of about 99.0%).
  k. Simpler manipulation and work-up (automatic MW oven).
  l. Higher selectivity of the reaction (extremely irreversible hydrazinolysis reaction*).*
  m. Increased atom economy and efficiency (just two reactants giving one final product with much higher overall yield in one step).
  n. Increased carbon and mass efficiency (100.0%).

### Procedures

#### General data and considerations

All reactions were performed with commercially available reagents. All chemicals (reagents and solvents) were of analytical grade, purchased from commercial suppliers, and were used as received without further purification. MWI for MW reaction was carried out in an unmodified domestic MW oven (*Samsung* type, model M1733N with T.D.S. property, and having a power level of 100–800 W) operated at 2.45 GHz. TLC was used to monitor the progress of all reactions and to check the purity of products, it was carried out on TLC silica gel 60 F_254_ plates (plates of aluminum sheets precoated with unmodified silica gel 60 F_254_ to a layer thickness of 0.20 mm, purchased from E. Merck, Merck Millipore Division or Merck Chemicals, Merck KGaA, Darmstadt, Germany) as the stationary phase using pet. ether/EtOAc/abs. EtOH (6:3:2, v/v/v) mixture as the mobile phase (the eluting solvent system) for monitoring the esterification reaction for the synthesis of **1nz**, while a mixture of 5% abs. MeOH in CH_2_Cl_2_ (dichloromethane or methylene chloride) was used as the mobile phase for monitoring the hydrazinolysis reaction for the synthesis of **2nz**, and the chromatograms spots were visualized and observed under the used UV light at wavelengths of 254 (mainly) and 366 nm to detect the produced components. Evaporation and concentration was carried out by using rotavap under reduced pressure. A lyophilizer (freeze dryer, model FD8-8T, SIM international, U.S.A.) was used for the lyophilizing purpose. Melting points (°C) of both the synthesized compounds were measured and recorded in open glass capillaries using *Fisher-Johns* melting point apparatus and were uncorrected. IR spectrum for **2nz** was recorded on *Nicolet*™ iS™ 10 Mid-Infrared (ThermoFisher Scientific) FT-IR spectrometer (υ in cm^−1^) using KBr (potassium bromide) disk at the Central Laboratory (Faculty of Pharmacy, Mansoura University, Mansoura, Egypt) (if the abbreviated word “Str.” is not mentioned, this means that the peak is weak to medium in intensity). ^1^H-NMR spectrum for **2nz** was recorded on *Varian Gemini*-300 spectrometer (Mercury-300BB "NMR300") at about 300 MHz using TMS as an internal standard at the Microanalytical Center (Faculty of Science, Cairo University, Cairo, Egypt) and its chemical shifts values (δ) were given in ppm downfield from TMS at a temperature of 30 °C using DMSO-*d*_6_ as a solvent. ^13^C-NMR spectrum for compound **2nz** was recorded also on *Varian Gemini*-300 spectrometer (Mercury-300BB "NMR300") at about 75 MHz using TMS as an internal standard at the Microanalytical Center (Faculty of Science, Cairo University, Cairo, Egypt) and its chemical shifts values (δ) were given in ppm downfield from TMS at a temperature of 30 °C using DMSO-*d*_6_ as a solvent. MS analysis for compound **2nz** was performed on *Shimadzu* Qp-2010 Plus at 70 eV and results were represented by *m*/*z* (Rel. Int. in %) at the Microanalytical Center (Faculty of Science, Cairo University, Cairo, Egypt). Elemental analyses for **2nz** were performed at the Microanalytical Center (Faculty of Science, Cairo University, Cairo, Egypt) in order to determine C, H, and N contents in % (they, all, were in full agreement with the calculated theoretical values).

#### Conv. synthesis of **1nz**

To a stirred solution of anhydrous gallic acid (0.1 mole, 17.012 g) in abs. EtOH (500 mL) in a round-bottomed flask, conc. H_2_SO_4_ (5 mL) was added. The resulted reaction mixture was refluxed with stirring at 90–100 °C for 15 hr. After reaction completion (i.e., no more water was being distilled off), the reaction mixture was cooled to R.T., then xss. EtOH was completely evaporated (distilled off) on a water bath, and the reaction mixture was allowed to cool to R.T. again. The residue remained was taken up in pure distilled H_2_O in a separatory funnel and the flask was rinsed with a few mL of pure distilled H_2_O which were also poured into the separatory funnel. The formed aqueous solution was extracted by shaking it vigorously with EtOAc for three times (3 × 250 mL; the upper organic layer was carefully taken each time while the lower aqueous layer was finally rejected), the combined organic layers (extracts) were returned to a pure dry separatory funnel to be subsequently shaken with a strong solution of NaHCO_3_ (sodium bicarbonate) until all the free gallic acid was removed (i.e., until no more effervescence), then they were washed with distilled H_2_O and brine (a highly conc. NaCl (sodium chloride) solution in H_2_O). The organic layer was taken and dried over anhydrous Na_2_SO_4_ (sodium sulfate) during filtration (directly into the pure dry round-bottomed flask of the rotavap), then the filtrate was conc. by evaporation in rotavap under reduced pressure, left in air to be completely dry to give a crude white solid mass of the product **1nz** which upon further purification by recrystallization from hexane, it gives the pure product **1nz** (16.508 g, yield ≈ 83.3% (as reported [17,18])) which is a white amorphous powder with M.P. = 148–150 °C (as reported [17,18]).

#### Synthesis of **2nz**

##### **2nz** was synthesized by the following two methods:

###### 1- Common conv. heating method (from **1nz**):

A mixture of **1nz** (0.1 mole, 19.817 g) and slight xss. 85% NH_2_NH_2_·H_2_O (0.11 mole; about 5.35 mL) was dissolved in abs. EtOH (about 300–350 mL, i.e., the least amount needed to make the reaction mixture a clear solution) in a round-bottomed flask, then the resulted reaction mixture was mixed gently and refluxed with stirring for 6 hr. The progress of the reaction was monitored on TLC plates. After reaction completion, the reaction mixture was cooled to R.T., then xss. EtOH was completely distilled off on a water bath, and the reaction mixture was allowed to cool to R.T. again. The reaction mixture was poured onto ice-cold water with stirring and the white mass or precipitate obtained was filtered, washed with distilled H_2_O several times, and dried under vacuum (or left in air to be completely dry) to give a crude pale white solid mass of the product **2nz** which was recryst. twice from DEE and abs. MeOH (1:1, v/v) and further purified by HPLC analysis (**2nz** was successfully obtained with very excellent purity of about 99%; see details later) to give about 16.574 g (yield = 90.0%) of the pure **2nz** (pale white to buff fine powder) with M.P. = 294–296 °C.

###### 2- New green solvent-free one-pot MW-assisted method (from gallic acid)

A mixture of gallic acid (0.01 mole, 1.7012 g) and slight xss. 85% NH_2_NH_2_·H_2_O (0.012 mole; about 0.584 mL) was taken in a 150-mL conical flask, the resulted paste of the reaction mixture was well mixed, then the flask was covered with aluminum foil and subjected to intermittent MWI at intervals of 30 sec for 2 min (i.e., 4 intervals of 30 sec) at a power level of 800 W. The progress of the reaction was monitored on TLC plates till it was over after the fourth interval. After reaction completion, the reaction mixture was cooled to −20 °C and then it was lyophilized at −50 °C. The white product obtained was washed with distilled H_2_O several times and dried under vacuum (or left in air to be completely dry) to give a crude pale white solid mass of the product **2nz** which was recryst. twice from DEE and abs. MeOH (1:1, v/v) and further purified by HPLC analysis (**2nz** was successfully obtained with very excellent purity of about 99%; see details later) to give about 1.808 g (yield = 98.2%) of the pure **2nz** (pale white to buff fine powder) with M.P. = 294–296 °C.

• **HPLC analysis for separation of2nzand determination of its crude product purity:**

**Fig. 1:**
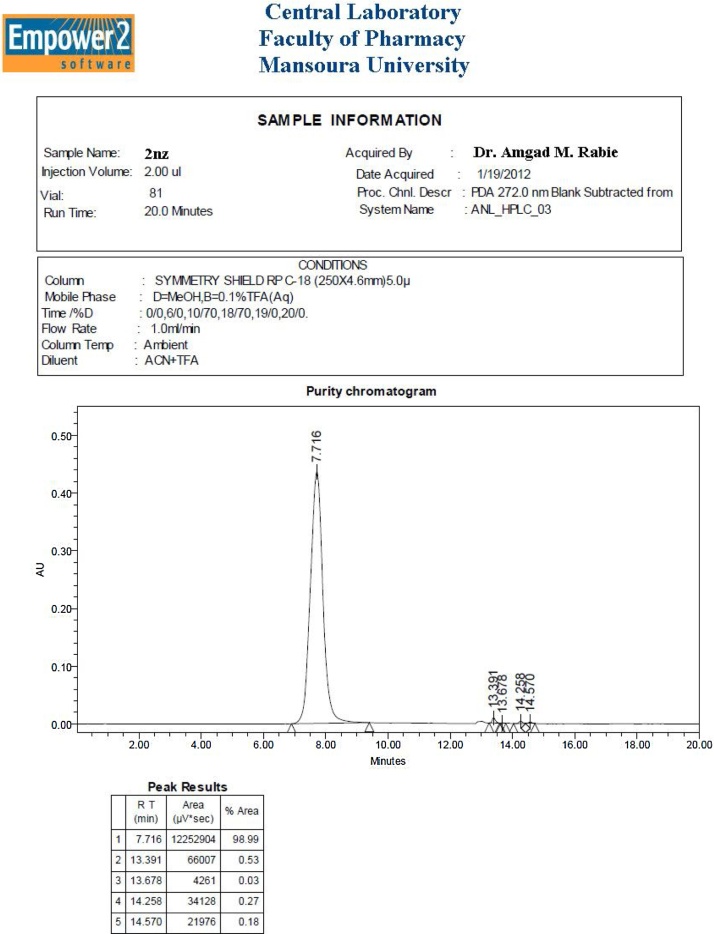
A scanned copy of the HPLC chart of analysis for compound **2nz** obtained in the present work (including sample information, analytical conditions, purity chromatogram, and peak results).

For these two purposes, the following HPLC analytical conditions were used (see Fig. 1, shown below):

•
  ○ *Column Type: SYMMETRY SHIELD RP C18 (250 × 4.6 mm; 5 μm; equipped with Empower 2 software).*
  ○ *Column Temperature: Ambient.*
  ○ *Mobile Phase Constituents: A = MeOH; B = 0.1% TFA in H_2_O.*
  ○ *Diluent Constitution: ACN + TFA.*
  ○ *Injection Volume: 2 μL.*
  ○ *Flow Rate: 1 mL/min.*
  ○ *Run Time: 20 min.*
  ○ *Gradient Solvent System (Time (min)/A (%)): 0/0; 6/0; 10/70; 18/70; 19/0; 20/0.*
  ○ *Wavelengths for UV Detection: 220 & 272 nm.*

### Data, value, and validation

#### Synthetic scheme of **2nz**

The synthetic scheme of the present research (Scheme 1, shown above) was adopted for the synthesis of the target hydrazide **2nz** which is either conventionally synthesized from the corresponding ester **1nz** (which is firstly synthesized from gallic acid by conv. esterification), or directly synthesized from the corresponding gallic acid by MW-assisted method without passing through the esterification step. This scheme provides the best synthetic route for **2nz**.

#### Comparative assessment study of MW method versus conv. method of synthesis of **2nz**

This comparison (Table 1, shown below) demonstrates the detailed point-by-point superiority of the new green one-step MW-assisted heating procedure over the traditional hazardous two-step conv. heating procedure for the efficient synthesis of the hydrazide **2nz**.

**Table 1:** Comparative assessment of conv. method versus MW method of synthesis of galloyl hydrazide (**2nz**) from gallic acid (anhydrous) using many of the various green chemistry matrices (measures and metrics) with their improvement degrees in the MW method relative to the conv. method.

Matrix	Conv. Method	MW Method	Improvement
*Overall Yield (%)*	90.0	98.2	8.2% increase (more productive method)
*Overall Heating Time*	21 hr (75600 sec)	120 sec	630 times less (time-saving method)
*Number of Steps*	Two (two different separate reactions)	One (one-pot method)	50% decrease (simpler and cheaper method)
*Solvent*	Present (in the second step)	Absent	Solvent-free or solventless greener method
*Alcohol for Esterification*	Present (in the first step)	Absent (no need for this step)	One-pot alcohol-free greener method
*Catalyst and Dehydrating Agent*	Present (conc. H_2_SO_4_ in the first step)	Absent (no need for this step or dehydrating agent)	More efficient simpler greener method
*Energy Consumption (KWhr)*^a^	42	0.02667	About 1575 times less (energy-saving method)
*E-Factor*^b^	4.8	0.2	95.83% decrease (in the total waste produced in Kg over the production of 1 Kg of the product)
*Atom Economy (%)*^c^	69.16	83.64	14.48% increase (in the greenness of the method)
*Atom Efficiency (%)*^d^	62.24	82.14	19.90% increase (in the efficiency of the method)
*Carbon Efficiency (%)*^e^	77.78	100.00	22.22% increase

a KWhr: Kilowatt-hour(s); energy consumption (KWhr) is equal to the power P (in watts or W) multiplied by time t (in hr) divided by 1000 W per kilowatt (KW), i.e., energy consumption (KWhr) = P (W) × t (hr) / 1000 (W/KW), where, in this present work, P of the used laboratory heater or hot plate, i.e., for conv. method, is 2000 W and adjusted P of the used MW oven, i.e., for MW method, is 800 W (P of MW oven for the synthesis of **2nz**).

b E-factor: Environmental factor, E-factor = mass of total waste (Kg) / mass of product (Kg), the value of this factor depends on one's definition of “waste’’, so it varies.

c Atom economy (%) = M.Wt. of product × 100 / (M.Wt. of reactant 1 + M.Wt. of reactant 2 + M.Wt. of reactant 3 + … etc.).

d Atom efficiency (%) = % overall yield × % atom economy × 100.

e Carbon efficiency (%) = mass of carbon in product × 100 / total mass of carbon present in all reactants (i.e., number of moles of product × number of carbons in product / [(number of moles of reactant 1 × number of carbons in reactant 1) + (number of moles of reactant 2 × number of carbons in reactant 2) + (number of moles of reactant 3 × number of carbons in reactant 3) + … etc.].

#### Peak results of purity chromatogram for HPLC analysis and purification of **2nz**

HPLC analysis is used for the separation of **2nz** from other related gallic/gallate derivatives that might be present in the crude **2nz** in extremely minute quantities after recrystallization process (not exceeding 1% of the total weight) as minor impurities (i.e., HPLC analysis is used for further purification and determination of purity of the crude **2nz** produced in this present work after recrystallization). For detection, separation, and determination (of their percentage in the total weight) of these closely related impurities from the crude **2nz**, preparative HPLC and RP techniques of chromatography were used (see Fig. 1, below, for the crude details of the conditions and results of this assay). Peak results of purity chromatogram for HPLC analysis of **2nz** are shown below in Table 2.

**Table 2:** Peak results for purity chromatogram of HPLC analysis of compound **2nz**.

Fraction Isolated	Run Time (min)	Area (%)
*I (****2nz****)*	7.716	**98.99**
*II (Related Impurity A)*	13.391	0.53
*III (Related Impurity B)*	13.678	0.03
*IV (Related Impurity C)*	14.258	0.27
*V (Related Impurity D)*	14.570	0.18

Results from Table 2 show that the percentage of pure **2nz** in crude **2nz** (obtained by both methods) is about 99% (the major fraction isolated) and the percentage of related compounds (the four fractions of impurities isolated) in it is only about 1% (i.e., the crude **2nz** obtained is about 99% pure).

#### Physicochemical characterization data of **2nz**

The separated highly pure **2nz** is a pale white to buff fine powder with a M.P. of 294–296 °C. It is mainly freely soluble in DMSO (but its solubility in other organic solvents differs from one to another according to the strength of the solvent).

#### Spectroscopic and microanalytical characterization data (structure elucidation) of **2nz**

**Fig. 2:**
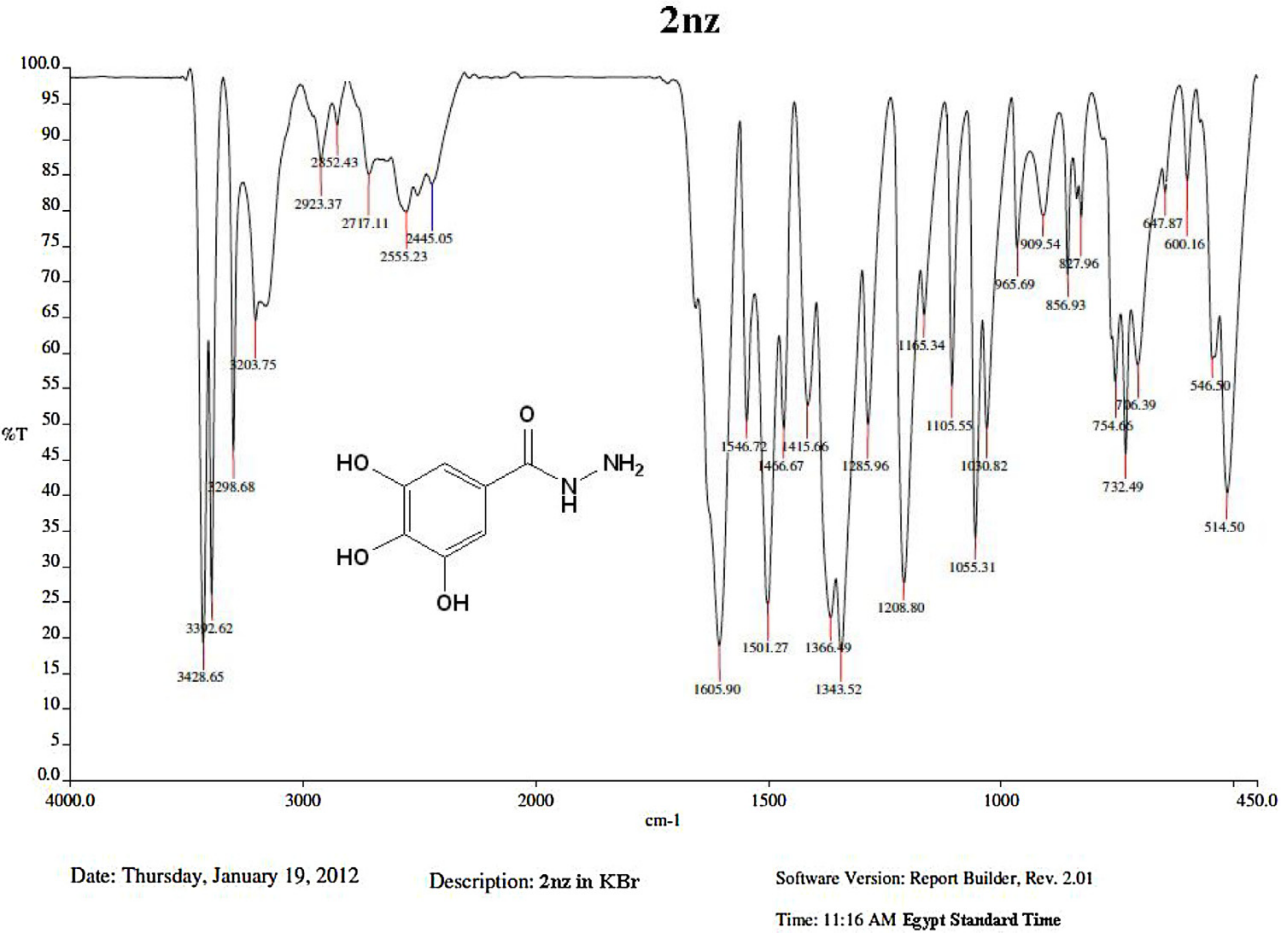
A scanned copy of the spectral chart obtained upon IR spectroscopic analysis of the sample of the compound **2nz**.

**Fig. 3:**
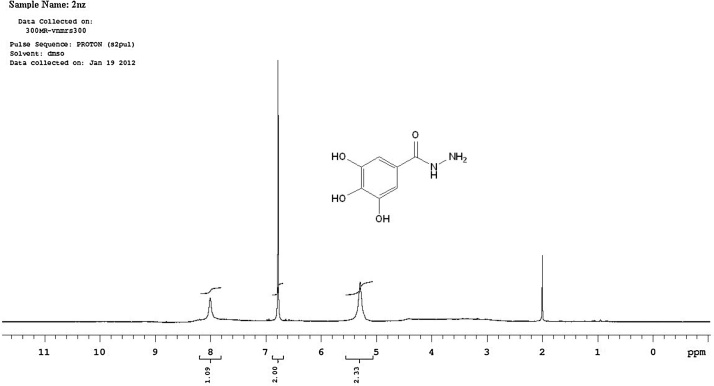
A scanned copy of the spectral chart obtained upon ^1^H-NMR spectroscopic analysis of the sample of the compound **2nz**.

**Fig. 4:**
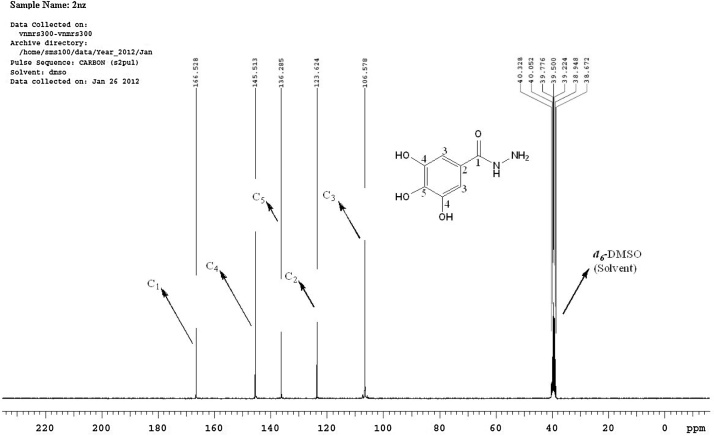
A scanned copy of the spectral chart (explained) obtained upon ^13^C-NMR spectroscopic analysis of the sample of the compound **2nz**.

**Fig. 5:**
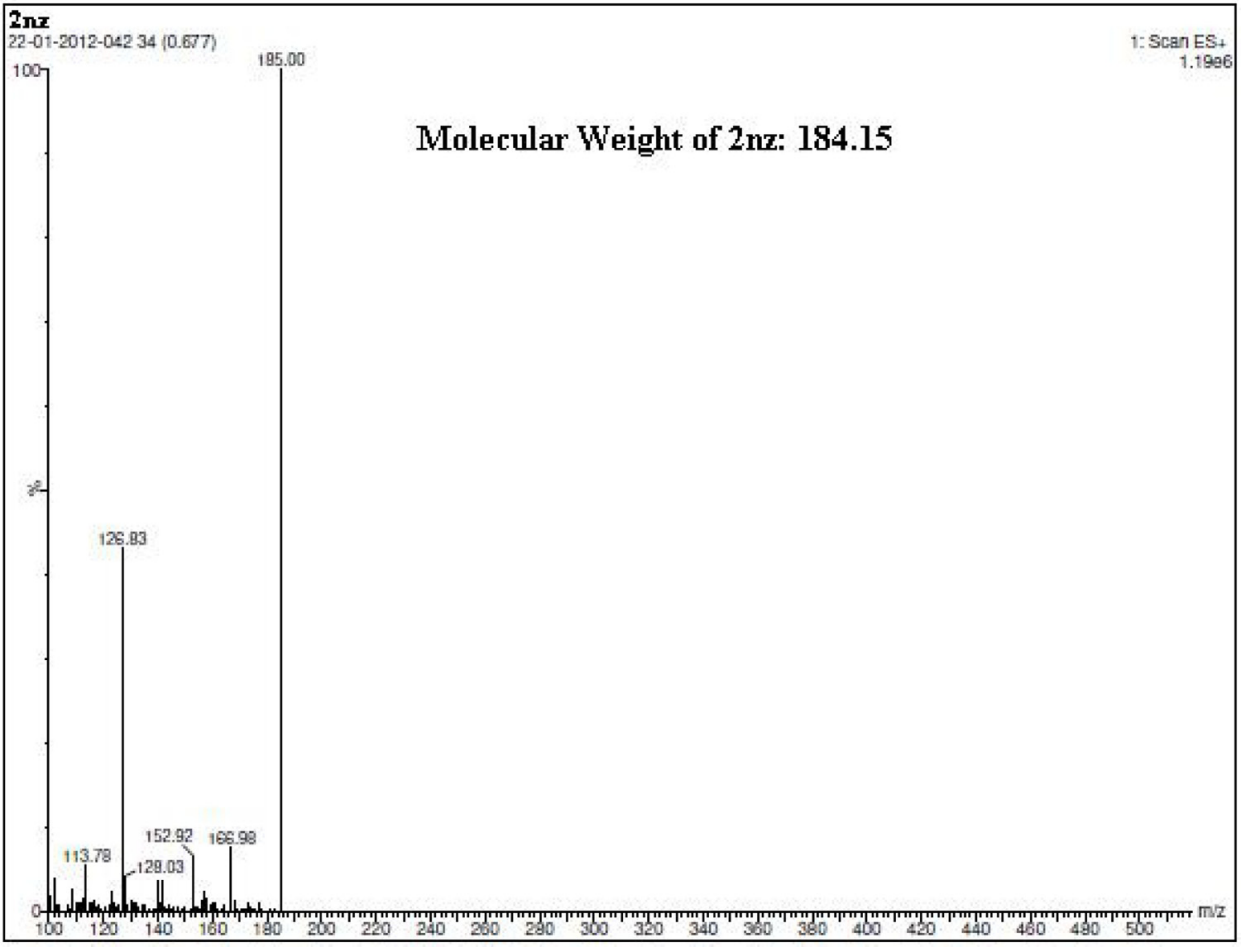
A scanned copy of the spectral chart obtained upon MS analysis of the sample of the compound **2nz**.

**Fig. 6:**
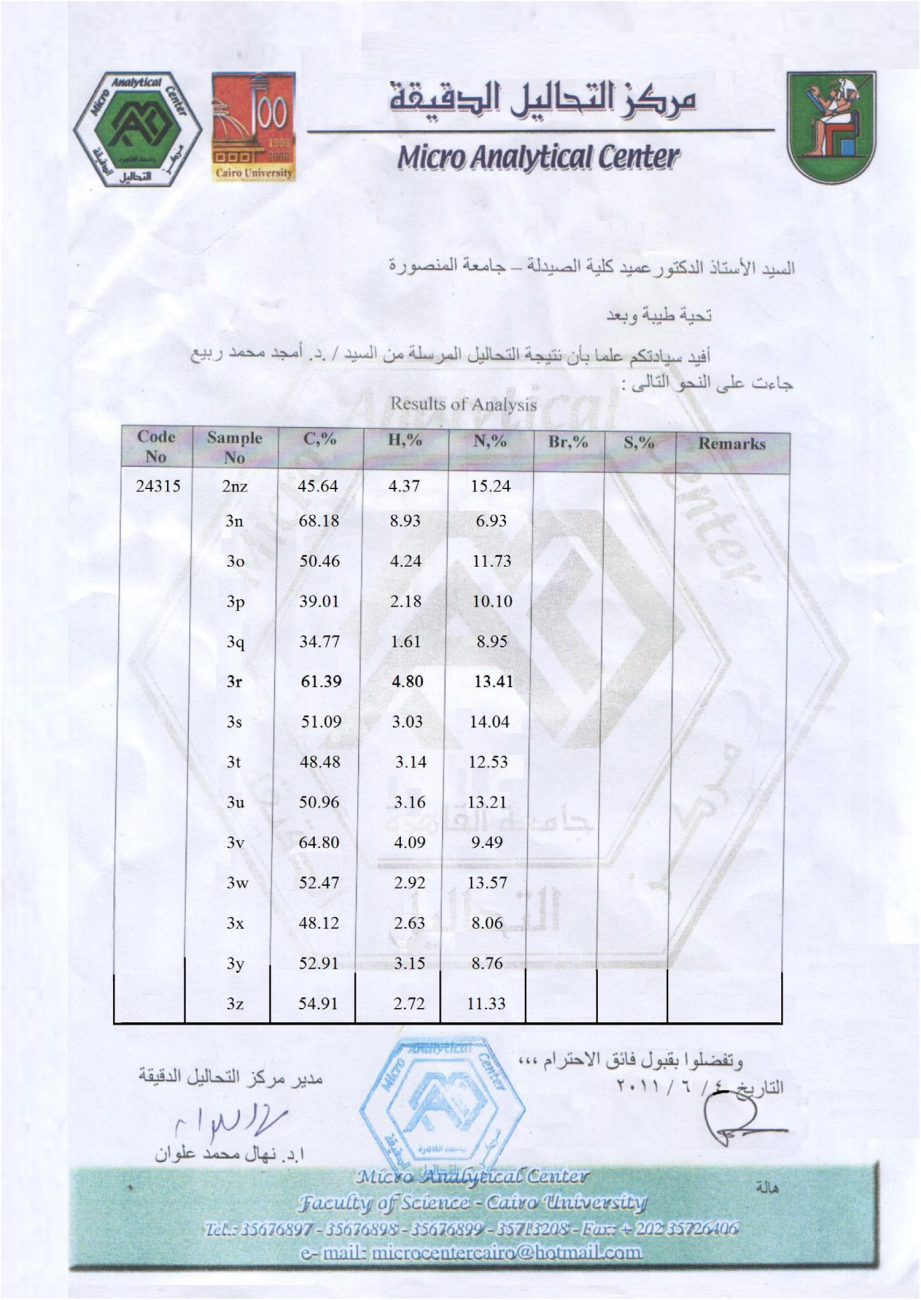
A scanned copy of the results of the elemental analyses (C, H, and N) of the sample of the compound **2nz** (only the first compound in the table).

The separated highly pure **2nz** was subjected to full spectroscopic and elemental analyses for identification and confirmation of its structure (see Fig. 2, Fig. 3, Fig. 4, Fig. 5, Fig. 6, below, for the crude details of the conditions and results of these assays). The collective IR, ^1^H-NMR, ^13^C-NMR, MS, and elemental (C, H, and N content) analyses data obtained for **2nz** are shown, respectively, below in Table 3.

**Table 3:** Spectroscopic and microanalytical characterization data of compound **2nz**.

## Declaration of Competing Interest

The author declares that he has no known competing financial interests or personal relationships that could have appeared to influence the work reported in this paper.
